# Feedback on the Implementation of a Rapid and Connectable Point-of-Care COVID-19 Antigen Test in an Emergency Department

**DOI:** 10.3390/diagnostics13233508

**Published:** 2023-11-22

**Authors:** Caroline Coulon, Anne-Sophie Bargnoux, Océane Jourdan, Vincent Foulongne, Anne-Marie Mondain, Anne-Marie Dupuy, Mustapha Sebbane, Jean-Paul Cristol

**Affiliations:** 1 Department of Biochemistry and Hormonology, University Hospital of Montpellier, 34295 Montpellier, France; 2 Department of Biochemistry and Hormonology, University Hospital of Montpellier, PhyMedExp, Institut National de la Santé et de la Recherche Médicale, Centre National de la Recherche Scientifique, University of Montpellier, 34295 Montpellier, Francejp-cristol@chu-montpellier.fr (J.-P.C.); 3 Department of Virology, University Hospital of Montpellier, 34295 Montpellier, France; 4 Centre de Ressources Biologiques, University Hospital of Montpellier, 34295 Montpellier, France; 5 Department of Emergency Medicine, University Hospital of Montpellier, 34295 Montpellier, France

**Keywords:** SARS-CoV-2, point-of-care, antigen test

## Abstract

Faced with the pandemic viral circulation of SARS-CoV-2, healthcare establishments have had to maintain an effective screening strategy in order to prevent nosocomial clusters. Automated antigenic tests appear to be a reliable and complementary alternative to RT-PCR (reverse transcriptase polymerase chain reaction) in order to optimize patient care in the emergency department. We report our experience of the deployment of the LumiraDx antigen tests on the LumiraDx platform, as well as the comparison of these tests’ results with the RT-PCR results on a population of patients sampled in the emergency department.

## 1. Introduction

In the context of the SARS-CoV-2 viral pandemic, healthcare establishments have been forced to consider an effective screening strategy to rapidly target positive cases and implement appropriate isolation measures to prevent the emergence of nosocomial clusters. Although reverse transcriptase polymerase chain reaction (RT-PCR) is the reference method and the only one capable of determining the variant responsible for the infection [1,2], automated and connectable antigenic testing platforms have emerged as a complementary alternative to reference molecular tests, reducing the time needed to deliver results to healthcare departments. The University Hospital of Montpellier (France) is a multisite hospital 2111-bed facility with over 300 emergency-room visits a day. One of the factors affecting the smooth running of emergency departments, particularly during epidemic waves, is the management of transfers of patients requiring hospitalization to downstream care services. Indeed, these departments are often equipped with double rooms, reinforcing the need for viral detection prior to referral. Initially, the non-contagiousness of patients prior to their transfer to the medical wards was confirmed on the basis of RT-PCR negativity alone. However, results could take between 3 h and 8 h, depending on the number of requests and the distance between the sampling site and the central laboratory. The evolution of recommendations on the use of antigenic tests prompted us to think about our screening strategy [3,4]. In this context, it seemed worthwhile to study the possibility of deploying a system that could be used directly in the care departments at the point of care (POC). At the height of the epidemic, in January 2022, the Montpellier University Hospital equipped itself with the LumiraDx SARS-CoV-2 Ag 12 min test on the LumiraDx platform, after evaluating the test’s analytical performances. However, a review of the advantages and disadvantages of this implementation highlighted the fact that healthcare teams concluded that the time taken to deliver the results was too long. In May 2022, LumiraDx launched a new version of the antigen test with a shorter turnaround time (5 min). Consequently, the first objective of this real-life study is to analyze the results of a concordance study between the two LumiraDx antigenic test references (12 versus 5 min test duration) and to compare these results with those obtained by RT-PCR on a population of patients sampled in emergency departments. The second objective is to provide feedback on the use of this analytical solution.

## 2. Materials and Methods

The RT-PCR test used in the central laboratory of virology is the Alinity m SARS-CoV-2 assay. The LumiraDx SARS-CoV-2 Ag test is a microfluidic immunofluorescence point of care (POC) assay for direct and qualitative detection of the SARS-CoV-2-specific nucleocapsid protein antigen in nasal and nasopharyngeal swab specimens. The LumiraDx SARS-CoV-2 Ag test provides results in 12 min. A more recently developed version, LumiraDx SARS-CoV-2 Ag Ultra test, allows for a reduction in dosing time to 5 min. The limit of detection (LoD) of the LumiraDx SARS-CoV-2 Ag Ultra test was evaluated by manufacturer by testing 20 replicates with concentrations at the tentative LoD. Based on this testing, the LoD for nasal swab samples was confirmed at 800 TCID50/mL. The test environment was controlled by a simplified calibration process (RFID chip) and quality controls whose frequency could be customized to suit the needs of the department. The marketed quality control reference included two ready-to-use reagents: a negative control and a positive control (LumiraDx SARS-CoV-2 Ag Quality Controls). The LumiraDx platform was connected to the laboratory’s IT system, allowing the results to be downloaded directly into the patient’s medical record.

Because of the low incidence and positivity rates at the start of the study (September 2022), participants (aged ≥18 years) were gradually recruited with a random sampling method between September 2022 and March 2023 from the emergency department of the University Hospital of Montpellier (France). These patients were sampled twice with nasopharyngeal swabs (one swab in each nostril). One swab was used for the LumiraDx antigen tests (12 min and 5 min) using the LumiraDx extraction buffer, and further processed and tested according to the LumiraDx SARS-CoV-2 Ag test product insert. The second swab followed the RT-PCR process.

An analysis of the sensitivity and specificity of the LumiraDx tests was performed for a Ct of 30, the contagiousness threshold defined by the French Scientific Council [5], and a Ct of 33, the threshold compatible with a significant viral excretion according to the French Society of Microbiology [6], whose recommendations are based on various European and international works [7,8,9].

## 3. Results

### 3.1. Cohort

There were 329 symptomatic or non-symptomatic adults (blind recruitment) included in the study. The median age of the participants was 51 (18–98) years old, with 47% being female. Among these patients, 31 patients were found to be “positive” using the RT-PCR test, regardless of the cycle threshold (Ct) value considered (Ct ranging from 15.8 to 38.2).

### 3.2. Results Presentation

An analysis of the sensitivity and specificity of the LumiraDx SARS-CoV-2 Ag tests is presented in Table 1 and Table 2. The Supplementary Materials present the details of the positive percent agreement for the LumiraDx tests with RT-PCR, depending on the Ct value considered (Tables S1 and S2).

The concordance study between the LumiraDx tests and RT-PCR considering the Ct threshold of 30 displayed a sensitivity of 100% for both the LumiraDx SARS-CoV-2 Ag test (12 min) and the LumiraDx SARS-CoV-2 Ag Ultra test (5 min). The concordance study between the LumiraDx tests and RT-PCR considering the Ct threshold of 33 displayed a sensitivity of 91.7% and 87.5% for the LumiraDx SARS-CoV-2 Ag test (12 min) and the LumiraDx SARS-CoV-2 Ag Ultra test (5 min), respectively.

For both antigenic tests, the specificity and positive predictive value were close for both of the Ct values considered (30 or 33). Moreover, for the negative predictive value at 30 Ct or 33 Ct, the two values used for contagiousness thresholds [5,6] were greater than 99%.

## 4. Discussion

The analysis of the sensitivity and specificity for the LumiraDx SARS-CoV-2 Ag tests shows that the LumiraDx system meets the performance requirements set by WHO, namely sensitivity ≥80% and specificity ≥97% when compared with a nucleic acid amplification test [10]. The performances of the LumiraDx SARS-CoV-2 Ag test (12 min) have already been studied [11,12,13,14], showing an excellent analytical sensitivity and specificity. Our study confirms these performances and, for the first time, demonstrates that the 5 min version of the antigenic test (LumiraDx SARS-CoV-2 Ag Ultra test) offers a similar performance.

The LumiraDx SARS-CoV-2 Ag test (12 min) already significantly reduces the time taken to deliver results to healthcare teams compared with an RT-PCR test, notably by eliminating the logistical steps involved in transporting the sample to the laboratory. The LumiraDx SARS-CoV-2 Ag Ultra test (5 min) offers a supplementary gain in reactivity. Healthcare teams have appreciated the reduced turnaround time for results, as this improves the fluidity of patient care in the emergency department. Furthermore, in the current context of developing point-of-care testing, it would have been interesting to precisely quantify the impact of the gain in analytical response time on the duration of patient’s stay in the emergency department.

In contrast with the antigen detecting rapid diagnostic by lateral flow test used in the past by the team, this connected platform ensures process traceability and, if required, provides health survey organizations with incidence and prevalence data in real time. On the other hand, regular quality controls ensure these tests are carried out safely.

In terms of organizational benefits, this unit test has the advantage of being easy to use, thus facilitating staff training. We trained around a hundred operators between the start and the end of the deployment period. The process is as follows. Briefly, simply elute the swab used for nasopharyngeal sampling into a tube containing an inactivating extraction buffer, then place a drop using the dropper cap on the microfluidic test strip. This process is compatible with use by qualified medical and/or paramedical staff, and enables a traceable result to be obtained while limiting identity−vigilance errors by reading the barcode on the patient’s hospitalization label.

The start-up period for this single-center study did not facilitate the recruitment of positive cases, which explains the size of the cohort and the low positivity rate. This may be explained by the inclusion of asymptomatic patients in the context of systematic screening upon entry to the emergency department in order to prevent nosocomial infections.

The existence of other tests marketed on this platform, in particular tests enabling the simultaneous qualitative detection of SARS-CoV-2, Influenza A, and/or Influenza B viral antigens, as well as the simultaneous detection of SARS-CoV-2 and respiratory syncytial virus (RSV) viral antigens, opens up new prospects in terms of screening strategies in epidemic periods in departments with a large turn-over of patients.

## 5. Conclusions

The LumiraDx SARS-CoV-2 Ag Ultra test appears to meet the sensitivity and specificity requirements for a screening test designed to exclude SARS-CoV-2 infection. The system’s connectability means that results can be traced in the computerized medical record. The analytical response time is suitable for use as a point-of-care COVID-19 antigen testing (POCT) to optimize patient flow within a healthcare establishment. The reduction in analytical response time compared with an RT-PCR test should translate into a reduction in time spent in the emergency department, which should reinforce the acceptability of this additional task to emergency staff.

## Figures and Tables

**Table 1:** Concordance table between LumiraDx tests and RT-PCR considering a Ct threshold of 30.

	LumiraDx SARS-CoV-2 Ag Ultra (5 min) versus RT-PCR (Ct 30)	LumiraDx SARS-CoV-2 Ag (12 min) versus RT-PCR (Ct 30)
Sensitivity	100%	100%
Specificity	98.4%	99.4%
Positive predictive value	80.8%	91.3%
Negative predictive value	100%	100%

Ct: cycle threshold; RT-PCR: reverse transcriptase polymerase chain reaction.

**Table 2:** Concordance table between LumiraDx tests and RT-PCR considering a Ct threshold of 33.

	LumiraDx SARS-CoV-2 Ag Ultra (5 min) versus RT-PCR (Ct 33)	LumiraDx SARS-CoV-2 Ag (12 min) versus RT-PCR (Ct 33)
Sensitivity	91.7%	87.5%
Specificity	99%	99.3%
Positive predictive value	84.6%	91.3%
Negative predictive value	99.3%	99%

Ct: cycle threshold; RT-PCR: reverse transcriptase polymerase chain reaction.

## Data Availability

The research data associated with this article are available on request from the corresponding author (Caroline Coulon: caroline-coulon@chu-montpellier.fr).

## Supplementary Materials

The following supporting information can be downloaded at: https://www.mdpi.com/article/10.3390/diagnostics13233508/s1, Table S1: Positive percent agreement of LumiraDx SARS-CoV-2 Ag Ultra test with RT-PCR depending on the Ct value considered; Table S2: Positive percent agreement of LumiraDx SARS-CoV-2 Ag test with RT-PCR depending on the Ct value considered.
