# Supplementary material for: Feedback on the Implementation of a Rapid and Connectable Point-of-Care COVID-19 Antigen Test in an Emergency Department
Source: Diagnostics (Basel). 2023 Nov 22;13(23):3508. doi: 10.3390/diagnostics13233508 (PMC10706489; doi:10.3390/diagnostics13233508)
Supplement: Supplementary file 1 [file diagnostics-13-03508-s001.zip › diagnostics-2609089-supplementary.pdf]

Supplementary Materials:

Table S1. Positive percent agreement of LumiraDx SARS-CoV-2 Ag Ultra test with RT-PCR depending on the Ct value considered.

**LumiraDx SARS-CoV-2 Ag Ultra (5 min) *versus* RT-PCR**		
**Grouping**	N	Positive percent agreement (sensitivity)
**Ct (all)**	30	74.2
**Ct ≤ 33**	24	91.7
**Ct ≤ 30**	21	100
**Ct ≤ 25**	13	100
**Ct ≤ 20**	6	100

Ct : cycle threshold / RT-PCR : reverse transcriptase polymerase chain reaction.

Table S2. Positive percent agreement of LumiraDx SARS-CoV-2 Ag test with RT-PCR depending on the Ct value considered.

**LumiraDx SARS-CoV-2 Ag (12 min)**		
**Grouping**	N	Positive percent agreement (sensitivity)
**Ct (all)**	30	71.0
**Ct ≤ 33**	24	87.5
**Ct ≤ 30**	21	100
**Ct ≤ 25**	13	100
**Ct ≤ 20**	6	100

Ct : cycle threshold / RT-PCR : reverse transcriptase polymerase chain reaction.
